# Living on the Edge: Contrasted Wood-Formation Dynamics in *Fagus sylvatica* and *Pinus sylvestris* under Mediterranean Conditions

**DOI:** 10.3389/fpls.2016.00370

**Published:** 2016-03-23

**Authors:** Edurne Martinez del Castillo, Luis A. Longares, Jožica Gričar, Peter Prislan, Eustaquio Gil-Pelegrín, Katarina Čufar, Martin de Luis

**Affiliations:** ^1^Department of Geography and Spatial Management, University of ZaragozaZaragoza, Spain; ^2^Department of Yield and Silviculture, Department of Forest Techniques and Economics, Slovenian Forestry InstituteLjubljana, Slovenia; ^3^Agrifood Research and Technology Centre of Aragon, Instituto Agroalimentario de Aragón (IA2), Unidad de Recursos ForestalesZaragoza, Spain; ^4^Department of Wood Science and Technology, Biotechnical Faculty, University of LjubljanaLjubljana, Slovenia

**Keywords:** xylogenesis, European beech, Scots pine, microcore, cambial activity, Moncayo Natural Park

## Abstract

Wood formation in European beech (*Fagus sylvatica* L.) and Scots pine (*Pinus sylvestris* L.) was intra-annually monitored to examine plastic responses of the xylem phenology according to altitude in one of the southernmost areas of their distribution range, i.e., in the Moncayo Natural Park, Spain. The monitoring was done from 2011 to 2013 at 1180 and 1580 m a.s.l., corresponding to the lower and upper limits of European beech forest in this region. Microcores containing phloem, cambium and xylem were collected biweekly from twenty-four trees from the beginning of March to the end of November to assess the different phases of wood formation. The samples were prepared for light microscopy to observe the following phenological phases: onset and end of cell production, onset and end of secondary wall formation in xylem cells and onset of cell maturation. The temporal dynamics of wood formation widely differed among years, altitudes and tree species. For *Fagus sylvatica*, the onset of cambial activity varied between the first week of May and the third week of June. Cambial activity then slowed down and stopped in summer, resulting in a length of growing season of 48–75 days. In contrast, the growing season for *P. sylvestris* started earlier and cambium remained active in autumn, leading to a period of activity varying from 139-170 days. The intra-annual wood-formation pattern is site and species-specific. Comparison with other studies shows a clear latitudinal trend in the duration of wood formation, positive for *Fagus sylvatica* and negative for *P. sylvestris*.

## Introduction

A forest community can prosper only on sites where the environmental conditions are within the niche volumes of each species (Reed and Clark, 1978). The distribution of different species is limited by a combination and interaction of biotic and abiotic factors (Mcinerny and Etienne, 2012); outside such conditions, the species cannot survive (Kearney, 2006).

The widespread forest species European beech (*Fagus sylvatica*) and Scots pine (*Pinus sylvestris*) have both high ecological relevance and economic values. European beech forests are spread all over central Europe, from central Poland, the south of Scandinavia and British Isles to the southernmost locations in the mountain ranges of Greece, Italy and Spain (Jalas and Suominen, 1973). Scots pine forests are distributed from the Alps to northeast Europe, covering all Scandinavia and Russia beyond 55° northern latitude (Jalas and Suominen, 1976). In the Mediterranean region are isolated patches of both species, climatically constrained by the warmer and drier conditions compared to the greater part of Europe. In these areas, extreme climatic events, such as summer droughts, heat waves or late frosts, restrict forest expansion on the edge of the distribution limit, leaving some populations isolated in mountain shelters.

Climate not only affects forest distribution but also tree growth. The study of cambial activity and tree-ring formation provides valuable information for understanding how trees respond to different climatic conditions (de Luis et al., 2011a; Gričar et al., 2014). In temperate ecosystems, climatic variability leads to an annual periodicity of cambial activity, with winter dormancy and an active period during the growing season.

Monitoring wood formation at the edge of a species’ distribution is therefore especially relevant, since these trees are most sensitive to limiting climatic factors and respond most distinctively to any change (Fritts, 1972; Gruber et al., 2010; González-González et al., 2014). Knowing how these species grow may help to predict the distribution of tree species in the context of the expected climate change scenarios (de Luis et al., 2011a). In particular, more extreme climatic conditions are expected to affect tree-species’ distribution (Richter et al., 2012; Eilmann et al., 2014).

Although the wood-formation patterns of *Fagus sylvatica* and *Pinus sylvestris* have been studied on different sites in Europe, studies along the western – southern distribution limits are still missing. Rossi et al. (2013) and Cuny et al. (2015), in comprehensive studies, compiled data on cambium phenology and wood-formation dynamics for several conifer species, including *P. sylvestris*, growing in different biomes. In Austria, *P. sylvestris* trees growing at xeric and dry-mesic sites were studied by Gruber et al. (2010), Oberhuber et al. (2011) and Swidrak et al. (2014). Similar studies were performed by Rathgeber et al. (2011a) and Cuny et al. (2012, 2014) in France, as well as by Seo et al. (2011) and Jyske et al. (2014) in Finland. These studies highlighted the plasticity of tree-ring formation of *P. sylvestris* in response to contrasting climatic conditions. Different key phenological dates showed distinct variability among study sites and years. It was shown that trees at northern sites initiate tree-ring formation later than trees at southern sites.

Compared to *P. sylvestris*, there is less information available on *F. sylvatica*. Cambial productivity of this species has been monitored at different sites and during several growth seasons in Slovenia by Čufar et al. (2008b) and Prislan et al. (2013) and also in Romania (Semeniuc et al., 2014). In addition, other studies have been performed for one growing season in the Netherlands (van der Werf et al., 2007), France (Michelot et al., 2012), Czech Republic (Vavrčík et al., 2013), and in north Germany (Schmitt et al., 2000). Previous studies indicated the importance of photoperiod and leaf phenology for the onset of xylem production, and the influence of climatic conditions in June, which was proved to be the most important month for wood formation (Čufar et al., 2014). Studies performed during several years showed that year-to-year variations in tree-ring formation can be explained by climatic conditions or environmental factors, although in some cases the relationship between variation in xylogenesis and weather conditions can be very complex (Prislan et al., 2013).

In order to better understand the growth adaptations and limitations of *F. sylvatica* and *P. sylvestris* at one of the Mediterranean edges of their distribution, we studied the dynamics of xylem-growth formation at one of the southernmost sites of the two species during three years. Cambium phenology (onset and cessation of cambial cell production) and the timing of xylem formation was compared between the two species and sites to evaluate the adaptation strategies under different environmental conditions. The duration of the xylogenesis was compared with data from other studies of the same two species performed all over Europe.

## Methodology

### Study Site

The study was carried out in the Moncayo Natural Park, a mountain area in the northeast of the Iberian Peninsula in the province of Zaragoza (41° 48′ 31″ N, 1° 49′ 10″ W). This natural park is considered as a biodiversity hotspot; the contrasting climate conditions along the altitudinal gradient of the mountain allow growth of various vegetation types, from Mediterranean to Eurosiberian species (Martinez del Castillo et al., 2015). This site is one of the southernmost forest stands in Europe for *F. sylvatica* and *P. sylvestris*. The mean annual temperature and average annual precipitation for the last 37 years were 11° and 710 mm, respectively [according to the Spain02 database (Herrera et al., 2012)].

Two pure stands of different altitudes were selected for each species, corresponding to the lower and higher altitudinal limits of *F. sylvatica* forest on this mountain. The low elevation site was located at 1180 m a.s.l. and the high elevation site at 1560 m a.s.l.

### Sample Preparation

The sampling of tissues for xylem-formation monitoring was performed biweekly from mid-March until late November from 2011 to 2013. At each sampling date, six trees were randomly selected per species and site in a sampling plot of around 50 m × 50 m. The selected trees were similar, healthy and dominant, with a stem diameter at breast height of 40–55 cm and an age of around 80 years for *P. sylvestris* and 35–50 cm and 120 years for *F. sylvatica*. From each tree, two microcores containing phloem, cambium and the last formed xylem growth ring were collected at breast height with a Trephor tool (Rossi et al., 2006a). The sample had a diameter of 2 mm and was up to 15 mm long. The sampling followed a helical arrangement around the stem to avoid wound effects from previous samplings, with sampling locations separated by at least 10 cm. After sampling, the microcores were immediately transferred in Eppendorf microtubes filled with formaldehyde-ethanol-acetic acid (FAA) fixative solution for one week and later stored in 70% ethanol.

The microcores were processed following the protocol described by Rossi et al. (2006a). The microcores were first dehydrated in a graded series of ethanol (70, 80, 90, and 100%) and infiltrated with D-limonene and paraffin using a Tissue Processor Leica TP1020. After infiltration, samples were embedded in paraffin blocks. Transverse sections of 8–10 μm thickness, depending on the species, were cut with a Leica RM 2245 rotary microtome. The sections were afterward stained with safranin and astra blue (Gričar et al., 2007; van der Werf et al., 2007; Prislan et al., 2013), mounted in Euparal and examined with a Nikon Eclipse E800 light microscope equipped with polarized light mode.

### Xylem Phenology Measurements and Data Processing

For *F. sylvatica*, the width of the cell layers in the cambium was measured with the NIS Elements BR3 image analysis system. Moreover, the width of growth-ring increments and also the width of tissues containing xylem cells in various differentiation phases were measured, i.e., post-cambial growth (enlarging cells), cells undergoing secondary wall thickening and mature cells. For *P. sylvestris*, the cambial cells were counted as well as the xylem cells in the three different aforementioned phases.

Cambial activity was identified and interpreted within the context of the multi-seriate concept, that the vascular cambium comprises both the cambial initial cells and xylem and phloem mother cells (Plomion et al., 2001). Thin-walled cambial cells were identified based on their small radial dimensions compared to xylem and phloem cells in the enlarging phase (post-cambial growth), with larger radial dimensions. The polarized mode of the light microscope enabled the discrimination between enlarging cells and secondary wall-thickening cells as described in Rossi et al. (2006b).

The number of cells and the width of tissues in each phase varied between and within trees due to the variation of the tree-ring width around the tree circumference. The number of cells in the previous xylem ring was therefore counted for *P. sylvestris* to normalize the measurements according to Rossi et al. (2003). In the case of *F. sylvatica*, the normalization formula was adapted using width measurements instead of cell number, as described in Prislan et al. (2013).

Extreme values were filtered and xylem-formation dynamics was analyzed with the Gompertz function (Rossi et al., 2003). Cambium phenology and timing of xylem formation were assessed using R package CAVIAR (Rathgeber et al., 2011a,b). We defined: beginning and end of the enlarging phase (bE, cE), beginning and end of the thickening phase (bW, cW) and beginning of cell maturation phase (bM). All the dates were computed with a dedicated function using logistic regressions. Differences in the different xylogenesis phases were determined applying repeated measurements ANOVA analysis (de Luis et al., 2011a; Prislan et al., 2013). The effects of fixed factors such as species and sites and the effect of time were evaluated. The total duration of the xylogenesis period was calculated by subtracting the beginning of the enlarging phase from the cessation of the thickening phase. Mean xylogenesis duration was calculated to compare the results with other studies.

## Results

### Wood Formation

**FIGURE 1:**
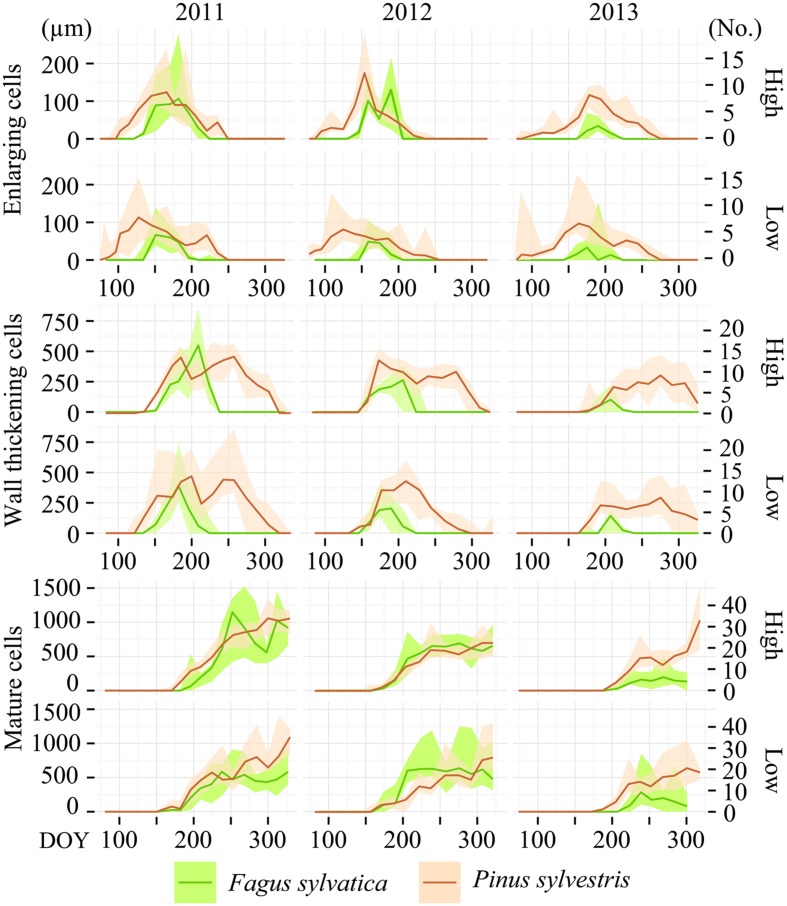
**Seasonal dynamics of xylogenesis phases in 2011, 2012, and 2013.** Amount (number for *Pinus sylvestris* and width of tissue for *Fagus sylvatica* expressed in micrometers) of enlarging cells, cells undergoing secondary wall formation and lignification, as well as mature cells in the currently formed growth rings on Moncayo low and high elevation sites. The lines represent the median and the blurred area the range between the 90th and 10th percentiles.

In all years, locations and species, the cambium was still dormant on the first sampling date in the last week of March. Despite site and annual variations in weather conditions, the different xylogenesis phases followed a common pattern during the growing season. The cell enlarging and cell-wall thickening curves follow a characteristic bell shape, while the cell maturation curve follows a sigmoid shape (**Figure 1**).

Overall, the onset of cell enlargement in *P. sylvestris* occurred between the last week of March and the first week of April, followed by the onset of the wall-thickening phase around 2 months later. The beginning of cell maturation occurred around the summer solstice, before the enlarging of cell ends in early September. Over more than 2 months, the currently forming tree-ring contained cells in different developmental stages. Completely mature xylem growth rings were observed in the first half of November.

Xylem-formation dynamics patterns differed between *F. sylvatica* and *P. sylvestris*. In *F. sylvatica* the onset of enlarging and wall-thickening phases occurred in the second half of May and in June, respectively. The beginning of the maturation process occurred from the last week of June to mid-July, followed by an immediate ending of the enlarging phase. Finally, the xylogenesis ended around mid-August.

### Phenology of Xylem Formation

**FIGURE 2:**
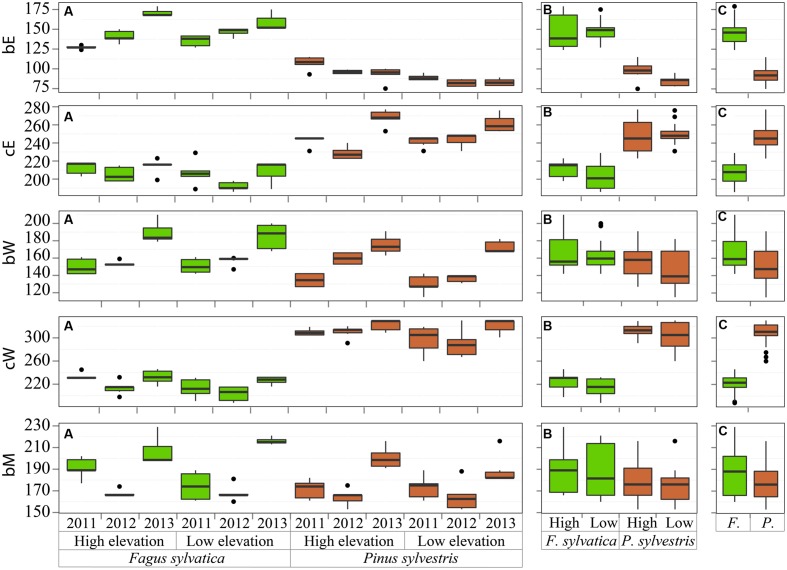
**Beginning and cessation of the cell enlargement (bE, cE) and cell-wall thickening phase (bW, cW) and beginning of maturation (bM) grouped by (A) sites, species and years, (B) sites and species and (C) species.** The central lines indicate the median value, vertical hinges indicate the first and third quartiles, error bars indicate the 95% confidence interval of the median and dots indicate values beyond the 95% confidence interval threshold.

The critical dates for the xylogenesis of *P. sylvestris* and *F. sylvatica* were summarized on three levels, shown in **Figure 2**. Dates significantly differed between the two species (ANOVA bE, cE, bW, cW: *p* < 0.001; bM: *p* = 0.005). The cell enlargement started first in *P. sylvestris*, around 31 March (DOY 90) and 50 days later, around 20 May (DOY 140) in *F. sylvatica*. Cessation of cell enlargement was observed between 9 July and 6 August (DOY 190-218) for *F. sylvatica* and in *P. sylvestris* between 10 August and 1 October (DOY 222-274). Cell-wall thickening and lignification began up to ca. one month earlier and ended around three months later in *P. sylvestris* than in *F. sylvatica*. The first mature cells were observed around 25 June (DOY 176) in *P. sylvestris* and around 7 July (DOY 188) in *F. sylvatica*.

The beginning of the enlargement phase was highly variable and significantly different among the years for both species at both high and low elevations (ANOVA species^∗^site: *p* < 0.001). In *F. sylvatica*, it began between 7 May and 20 June (DOY 127–171), with noticeable differences within years (**Figure 2**). In *P. sylvestris*, cell enlargement began between 23 March and 17 April (DOY 82–107). Although the variability was lower in the latter species, in 2011 there was a delay in the beginning of cell enlargement. Focusing on the elevation differences, cell enlargement started earlier at lower elevation in the *P. sylvestris* than in *F. sylvatica*. The end of this phase highly varied among years, whereby higher variability was observed between the years then among the sites for each species (ANOVA site: *p* = 0.31).

The onset of the secondary wall formation was highly variable (**Figure 2**); in all cases the cell-wall thickening phase started earlier in 2011 and later in 2013, followed by the same temporal pattern (within-subjects ANOVA time^∗^species^∗^site: *p* = 0.561) (**Figure 2**). The first mature cells (5%) were formed earliest in *P. sylvestris* at low elevation in 2012 (around June 4, DOY 155), and in 2011 (around 11 June, DOY 162) for *F. sylvatica*.

### Duration of the Growing Season

**FIGURE 3:**
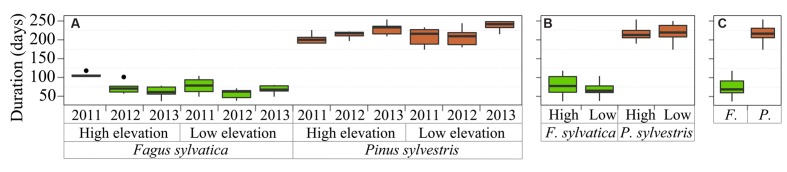
**Xylogenesis duration grouped by (A) sites, species and years, (B) sites and species and (C) species.** The box chart legend is as in **Figure 2**.

The total duration of the xylogenesis of *F. sylvatica* was significantly shorter than in *P. sylvestris* (ANOVA species: *p* < 0.001) (**Figure 3**). The cell production period during the three study years took 48–75 days for *F. sylvatica*, in contrast to *P. sylvestris*, with a growing period lasting from 140 to 170 days. Trees growing at low elevation had a longer growing period in the case of *P. sylvestris*, whereas the growing period of *F. sylvatica*, in contrast, was shorter at low elevation than at high elevation.

**FIGURE 4:**
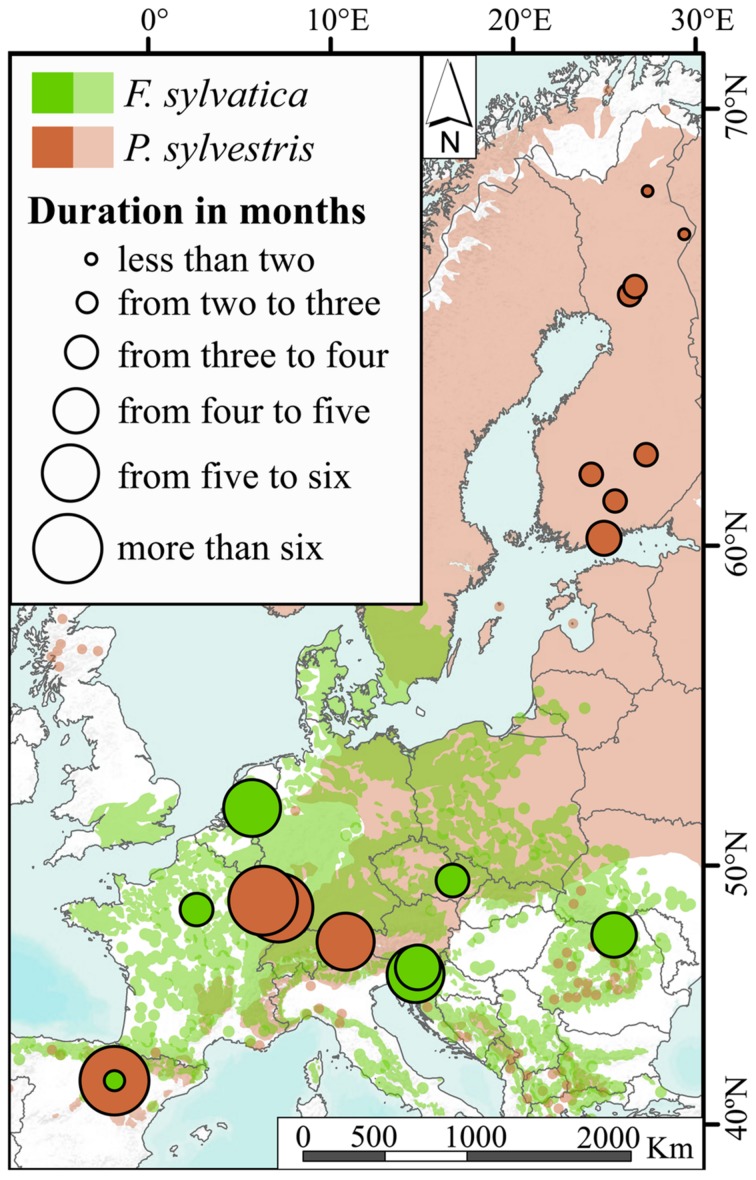
**Xylogenesis duration of *Fagus sylvatica* and *Pinus sylvestris* in Europe**.

**FIGURE 5:**
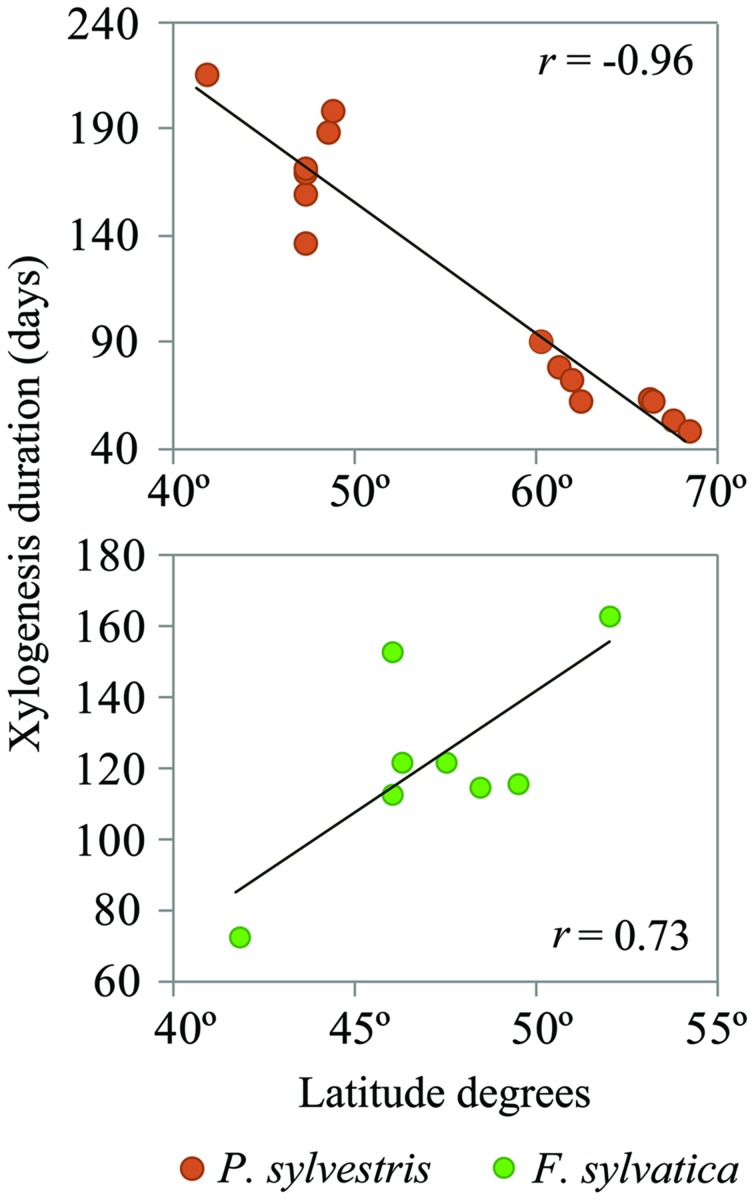
**Xylogenesis duration variations by latitude**. The black lines represent linear regressions: (*P. sylvestris*) *y* = -6.25x + 470.14, *P* < 0.001; (*F. sylvatica*) *y* = 7.28x -222.56, *P* = 0.010.

The mean duration of the xylogenesis was compared with other studies all over Europe (**Table 1** and **Figure 4**). In *P. sylvestris*, the duration of xylogenesis was shorter at high latitude and longer at low latitude, with a range of 49 days in Finland (Seo et al., 2011) to 217 days in Spain. In contrast, wood formation process in *F. sylvatica* was longer at high latitudes, over 163 days in the Netherlands (van der Werf et al., 2007) and only 67 days in Spain. In both species, the average duration of the xylem formation follows a linear pattern along the latitudinal range; however, whether it is directly or inversely proportional to the latitude depends on the species (**Figure 5**).

**Table 1:** Mean xylogenesis duration values of *Fagus sylvatica* and *Pinus sylvestris* from various wood-formation studies in Europe.

Species	Country	m a.s.l.	Long.	Lat.	Mean xylogenesis duration (days)	Reference
*Fagus sylvatica*	Spain	1560	-1.82	41.79	80	This study
	Spain	1180	-1.81	41.80	67	This study
	Slovenia	400	14.66	46.00	113	Čufar et al., 2008a
	Slovenia	400	14.66	46.00	153	Prislan et al., 2013
	Slovenia	1200	14.80	46.26	122	Prislan et al., 2013
	Romania	850	25.55	47.48	122	Semeniuc et al., 2014
	France	120	2.66	48.41	115	Michelot et al., 2012
	Czech Republic	630	16.70	49.46	116	Vavrčík et al., 2013
	Netherlands	50	5.71	51.98	163	van der Werf et al., 2007

*Pinus sylvestris*	Spain	1560	-1.82	41.79	215	This study
	Spain	1180	-1.81	41.80	217	This study
	Austria	750	10.84	47.23	137	Gruber et al., 2010
	Austria	750	10.84	47.23	160	Gruber et al., 2010
	Austria	750	10.84	47.23	170	Oberhuber et al., 2011
	Austria	750	10.84	47.23	172	Swidrak et al., 2014
	France	643	7.15	48.48	189	Cuny et al., 2012, 2014
	France	270	6.32	48.74	199	Rathgeber et al., 2011a
	Finland	60	25.00	60.20	91	Jyske et al., 2014
	Finland	120	25.60	61.20	79	Jyske et al., 2014
	Finland	181	24.30	61.90	73	Jyske et al., 2014
	Finland	110	27.30	62.40	63	Jyske et al., 2014
	Finland	140	26.40	66.20	64	Jyske et al., 2014
	Finland	140	26.70	66.30	63	Seo et al., 2011
	Finland	390	29.40	67.50	54	Jyske et al., 2014
	Finland	300	27.40	68.30	49	Seo et al., 2011

## Discussion

### Dynamics of Xylogenesis

Tree growth is largely affected by different climatic conditions, which become more limiting in adverse climatic conditions, such as in a Continental Mediterranean climate (Camarero et al., 2010; de Luis et al., 2011a; Pasho et al., 2012). Different tree species are differently affected by climate: e.g., evergreen or deciduous species, or early-successional or late-successional species. In this context, previous studies suggest that evergreen species adapt better to Mediterranean environmental and climatic conditions than deciduous species (Blumler, 1991), while early-successional species adopt riskier life strategies (Körner and Basler, 2010), making them more adaptive but also more vulnerable to the highly variable Mediterranean climate. These differences may trigger a different phenology of xylem formation. Our results suggest that *F. sylvatica* and *P. sylvestris* respond differently to local Mediterranean conditions. Accordingly, the phenology of xylem formation was significantly different between the two species, the period of all *P. sylvestris* developmental phases being significantly longer.

Our results demonstrate that Mediterranean climate has less impact on *P. sylvestris* than on *F. sylvatica*, despite this early-successional condition. The *Pinus* genus has been established as very plastic and capable of adapting its growth to changing climatic conditions (Camarero et al., 2010; de Luis et al., 2011a; Novak et al., 2013; Vieira et al., 2014) and the bimodal growth pattern as an adaptation to Mediterranean climate has been frequently described (Camarero et al., 2010; Campelo et al., 2015). Specifically, *P. sylvestris* has recently been determined as a plastic species in the Mediterranean area (Sánchez-Salguero et al., 2015).

Several studies performed on *F. sylvatica* under Mediterranean conditions have highlighted the growth limitation due to summer high temperatures and drought (Robson et al., 2013; Rasztovits et al., 2014; Chen et al., 2015; Rozas et al., 2015). In addition to climatic constrictions, *F. sylvatica* trees are more limited during the year in terms of plasticity because, with the activation of a leaf senescence mechanism, trees inexorably enter a dormant period. Despite this, our results reveal differences in the altitudinal gradient in agreement with the results shown in Prislan et al. (2013): who found similar patterns but different timing in two *F. sylvatica* forests with different climatic regimes.

The most striking result of the present study is the great differences in growth patterns among the years, highlighting a plastic response of radial growth in *F. sylvatica*, similarly as in *P. sylvestris.*

### Occurrence of Xylem Phenology

High variability in xylem phenology between years and sites demonstrates high plasticity of the species. The timing of different developmental phases significantly varied between the two species. Even though the variability of the critical dates was high among years and sites, the most remarkable disparity was found between the two tree species.

Xylogenesis, starting with cambial division and cell enlargement, is triggered by an increase in air temperature in spring. Several studies have demonstrated this positive relationship (Rossi et al., 2008; Prislan et al., 2011; Vieira et al., 2014), which has also been supported by stem heating experiments (Gričar et al., 2007; Begum et al., 2010). Under the same climatic conditions, we showed a difference in the onset of xylogenesis between the two species of over 50 days, especially in 2013, when the difference was about 72 days at both elevations. These differences suggest that climatic conditions for the onset of xylogenesis are species-specific. Moreover, the same weather conditions resulted in a completely different response of the tree species in terms of the temporal dynamics of xylogenesis, as can clearly be seen in 2011, when *F. sylvatica* started earlier than in the other two study years, while *P. sylvestris* showed the latest onset of growth in the same year.

The end of the cell-wall thickening phase seems to be a key date, since it defines the end of xylogenesis. Overall, the thickening phase in *P. sylvestris* continued until mid-November, whereas in *F. sylvatica* it ceased in mid-August, i.e., about three months earlier. Mild temperatures during early autumn may result in an extension of the growing period for *P. sylvestris* but not for *F. sylvatica*, since by that time leaf senescence has also already started. Cessation of cell-wall thickening was first observed in the lower part of the mountain in both species, as was similarly reported by Moser et al. (2009) and Oladi et al. (2011). This indicates that the end of xylogenesis is possibly influenced by temperature as well as the length of the photoperiod, as proposed by Plomion et al. (2001).

A common pattern is an evident delay in all developmental phases in 2013, except the beginning of wood formation in *P. sylvestris*. This may be explained by the late frost that occurred in 2013, after the onset of cell enlargement in *P. sylvestris*. This event presumably affected the temporal dynamics of wood formation in both species, although with different magnitudes. Menzel et al. (2015) showed that spring late frost events cause considerable damage in *F. sylvatica*. Nonetheless, a more detailed study of the climatic-growth relationship would be needed to confirm this hypothesis.

### Growing-Season Length under Mediterranean Conditions

Although both species are growing at their southern distribution limit and, consequently, their radial growth is somewhat constrained, the duration of the growing period of the two species significantly varied. The duration of xylogenesis highlights the differences between the species; it was two months for *F. sylvatica* and more than five months for *P. sylvestris*. Because of the early start and late end, *P. sylvestris* on Moncayo showed the longest xylogenesis duration in this species recorded in the various studies to date. Moreover, the shortest xylogenesis duration for *F. sylvatica* was also captured on this mountain.

Cambial resumption in *P. sylvestris* occurred earlier than on numerous Central European sites (Gruber et al., 2010; Oberhuber et al., 2011; Cuny et al., 2012, 2014; Swidrak et al., 2014), which could be explained by the warmer spring in Moncayo. These results are also in accordance with observations of Rossi et al. (2013) on various sites and Jyske et al. (2014) in Finland. On the other hand, the end of xylogenesis occurred later at Moncayo and resulted in a longer duration of xylogenesis than in other places in central and northern Europe. An extension of the growing season has also been described for other pine species, such as *P. halepensis* (de Luis et al., 2011b) or *P. pinaster* (Vieira et al., 2014). The extension of xylogenesis could be caused by the Mediterranean mild temperatures, despite water restrictions. However, in *F. sylvatica*, the results showed the opposite response, the beginning was later than in colder locations in Europe, such as in France (Michelot et al., 2012), Slovenia (Čufar et al., 2008b; Prislan et al., 2013), Romania (Semeniuc et al., 2014), Czech Republic (Vavrčík et al., 2013) and the Netherlands (van der Werf et al., 2007). This indicates that warmer and drier conditions at Moncayo negatively affect the duration of xylogenesis in *F. sylvatica*.

## Conclusion

It appears that the temporal dynamics of xylogenesis is considerably different in *F. sylvatica* than in *P. sylvestris* growing at the edge of their southern spatial distribution. This shows that intra-annual radial growth patterns in the studied species are differently affected by the Mediterranean conditions. The annual variation of the critical xylogenesis dates indicates a high species-specific plasticity for adapting to changing climatic conditions. As a result, the period of xylogenesis in *F. sylvatica* was around 2 months, while for *P. sylvestris* it was more than 5 months. Our findings are in accordance with our hypothesis of contrasting growth strategies and adaptations of the two species at the edge of their spatial distribution.

Furthermore, we compared our observations with those of other authors working on the same two species in different climatic environments, especially along a latitudinal range. A clear north–south trend was found in the xylogenesis duration over the distribution range of both species. *P. sylvestris* showed a positive xylogenesis duration trend on southern locations. *F. sylvatica*, in contrast, showed a shorter xylogenesis duration in the south of Europe than that shown in northern locations. These findings demonstrate that a deciduous and late-successional species such as *F. sylvatica* is negatively affected by Mediterranean climatic conditions, resulting in a shorter xylogenesis, whereas in the evergreen and early-successional *P. sylvestris*, xylogenesis is shown to be longer in Mediterranean environments.

## Author Contributions

EMDC did the fieldwork, wrote the paper and together with ML carried out the statistical analysis and prepared the figures. ML and LA developed the idea of this work and get the necessary funds. EMDC together with KČ, JG, and PP did the laboratory work and measurements. EG-P and KČ provided facilities and material support and gave technical support during laboratory work. All authors without exception helped to improve the work, specially the discussion and conclusions.

## Conflict of Interest Statement

The authors declare that the research was conducted in the absence of any commercial or financial relationships that could be construed as a potential conflict of interest.
